# Clinical evaluation and regression test of a commercial deep‐learning auto‐segmentation model

**DOI:** 10.1002/acm2.70824

**Published:** 2026-09-23

**Authors:** Roya Barati, Jingwei Duan, Song Gao, Yao Zhao, Xinru Chen, Cenji Yu, Zhan Xu, Moghadaseh Khaleghibizaki, Fredrik Lofman, Jared Ohrt, Laurence Court, Peter Balter, Jinzhong Yang

**Affiliations:** ^1^ Department of Radiation Physics University of Texas MD Anderson Cancer Center Houston Texas USA; ^2^ Department of Radiation Oncology Baylor College of Medicine Houston Texas USA; ^3^ Mayo Clinic Rochester Minnesota USA; ^4^ Graduate School of Biomedical Sciences The University of Texas MD Anderson Cancer Center UTHealth Houston Houston Texas USA; ^5^ RaySearch Laboratories AB Stockholm Sweden

**Keywords:** auto‐segmentation, commercial software, deep learning, quality assurance

## Abstract

**Background:**

Commercial deep‐learning segmentation (DLS) tools are increasingly used in clinical practice. Software updates may alter segmentation performance, highlighting the need for systematic clinical evaluation before implementation.

**Purpose:**

This study presents our experience in clinically evaluating and regression‐testing a RayStation DLS model with the aim of guiding commissioning and routine quality assurance of commercial DLS tools in clinical environments.

**Methods:**

U‐Net DLS model for normal‐tissue structures, originally commissioned in RayStation version 11B, was regression‐tested after upgrading to 2024A. Previously commissioned CT datasets for head and neck (*n* = 18), thorax (*n* = 18), male pelvis (*n* = 18), and breast (*n* = 21) were re‐evaluated in RayStation 2024A for regression testing following the software upgrade, while 20 new abdominal cases were included for initial commissioning. Clinical segmentations served as the reference standard. Geometric performance was evaluated using Dice Similarity Coefficient (DSC), 95% Hausdorff Distance (HD95), and Mean Surface Distance (MSD). Dosimetric evaluation used the original clinical treatment plans to compare Dmean and Dmax between DLS and clinical contours, normalized to prescription dose; breast was excluded from dosimetric analysis because its DLS model was not commissioned for clinical use.

**Results:**

Regression testing showed that, in comparison with the previously commissioned data, mean [± standard deviation (SD)] DSC values were 0.81 ± 0.09 for the head and neck, 0.84 ± 0.13 for the thorax, and 0.89 ± 0.06 for male pelvis. Corresponding MSD values were 0.83 ± 0.34 mm, 2.47 ± 1.81 mm, and 1.76 ± 0.68 mm; and HD95 values were 4.75 ± 2.38 mm, 8.45 ± 3.38 mm, and 13.21 ± 7.05 mm. Significant paired differences between versions were limited to selected structures, including the mandible, cochlea, and eye in head and neck; lungs and esophagus in thorax; and prostate in the male pelvis (*p* < 0.05). Breast segmentation results showed low performance (DSC 0.66 ± 0.15, MSD 4.8 ± 1.93 mm, and HD95 22.2 ± 9.6 mm) compared to clinical reference, so the breast structure models are not recommended for clinical use. Abdominal structures (RayStation 2024A vs clinical) had DSC 0.95 ± 0.014; MSD 0.82 ± 0.32 mm; and HD95 5.4 ± 2.9 mm. The mean dose differences between DLS and clinical segmentations were 0.03 Gy for the head and neck, –0.92 Gy for the thorax, 0.27 Gy for the male pelvis, and 0.02 Gy for the abdominal sites, and the corresponding maximum dose differences were 10.96 Gy, 13.23 Gy, 3.39 Gy, and 6.31 Gy.

**Conclusions:**

We described a procedure for practical quality assurance of auto‐segmentation tools for clinical use after periodic software upgrades. Regression testing of head and neck, thorax, and male pelvis sites showed consistent performance after the software upgrade and the DLS models for abdominal site were commissioned in this study for clinical use; while the DLS models for breast site were not recommended.

## 1 INTRODUCTION

Accurate delineation of organs at risk (OARs) remains a cornerstone of effective radiotherapy planning. Traditional clinical segmentation is labor‐intensive and subject to considerable inter‐observer variability.^1^, ^2^, ^3^ As the number of recognized OARs increases, and linkage with radiation side effects recognized, the additional complexity of the workload for treatment planning could compromise treatment consistency and outcomes.^4^, ^5^ This burden is further amplified in adaptive radiotherapy, where repeated delineation of clinical target volumes (CTVs) and OARs may be required on a daily basis. Recent advancements in artificial intelligence (AI) technologies have drastically changed the way OARs are delineated for radiotherapy planning.^6^, ^7^, ^8^ Deep learning (DL)‐based auto‐segmentation tools are emerging that show promise for greater accuracy, faster processing, improved efficiency, and enhanced consistency in auto‐segmentation compared with traditional segmentation approaches.^9^, ^10^ Nowadays, commercialized DL segmentation (DLS) tools are widely available for clinical use to automatically delineate OARs in radiotherapy planning. The domain shift between the training environment and real‐world settings, including both aleatoric uncertainty and epistemic uncertainty, can introduce suboptimal performance.^11^ Common clinical drivers include anatomic variability and differences in clinical practice, such as institutional protocols and guideline‐dependent contouring conventions. Reports have shown that even with careful training, unacceptable contours can still occur.^3^, ^12^ Given the crucial role of contouring in radiotherapy, rigorous and comprehensive evaluation of the clinical acceptability of any DLS models is essential before their clinical implementation.^13^, ^14^

In addition, the increasing number of DLS tools becoming available presents new challenges in safely using these tools in clinic routines, since no standard procedures are available to guide clinicians. Concerted efforts have been invested in medical physics to develop proper procedures for commissioning and routine quality checks of contouring tools.^15^, ^16^ Different institutions have their own standards, but no consensus standard procedure is available in the community. We present our approach with the hope of facilitating discussion on the development of standard procedures.

This study presents our approach in the use of regression testing to commission the DLS models within RayStation, when their software was upgraded from version 11B to 2024A. We validated the previously commissioned anatomic sites and also commissioned the new anatomic sites that were included in the upgraded 2024A version. Our experience may help in establishing standard procedures for routine quality assurance (QA) of DLS models.

## 2 MATERIALS AND METHODS

### 2.1 Patient data

This retrospective study was approved by the appropriate institutional review board. A total of 95 previously treated patients from five anatomic sites were retrospectively analyzed: head and neck (18), thorax (18), male pelvis (18), abdomen (20), and breast (21). Head and neck, thorax, male pelvis, and breast datasets were previously used to commission the DLS models in RayStation 11B and were re‐evaluated in 2024A, while the abdominal dataset was newly commissioned in this study. Clinical segmentations served as the reference standard for evaluation and were obtained from treated patient plans that had been approved by attending physicians. One experienced physicist reviewed the contours for integrity, and no contour editing was performed during this review process. The clinical contours were approved by a tumor board of multiple attendings within the same subspecialty, thereby minimizing inter‐observer variability of the clinical reference contours. All CT images had a resolution of 1 × 1 × 2.5 mm, with site‐specific acquisition protocols from either a Philips Brilliance Big Bore CT scanner or a Siemens SOMATOM Definition Edge CT scanner. All CT images have no contrast‐enhancement, except the abdominal CTs. The thorax images were average 4‐dimensional CT (4DCT) scans, and the abdomen images were contrast‐enhanced breath‐hold scans. For breast patients, all patients with right‐sided breast cancer had a free‐breathing scan, and all patients with left‐sided breast cancer had a deep‐inhaled breath‐hold scan. All cases are randomly selected from our clinical database with pre‐operative anatomy during treatment. They were not intentionally selected to include or exclude image artifacts, anatomical abnormalities or challenging cases. Treatment technique is either intensity modulated radiation therapy (IMRT) or volumetric modulated arc therapy (VMAT). Stereotactic body radiation therapy (SBRT) techniques were also included based on dose prescription.

### 2.2 DLS models

RaySearch Laboratories provides pre‐trained DLS models for CT images, trained separately per anatomic site (head and neck, thorax, breast, thorax‐abdomen, male pelvis) using a 3D U‐Net architecture (technical details in a published white paper).^17^, ^18^ The models are fully integrated into RayStation treatment planning system, so segmentation is run directly in the planning workflow and contours are used after an accuracy check. Version 11B models were previously commissioned in November 2022 for head and neck, thorax, and male pelvis; the breast model was tested but not commissioned due to low performance.^12^

In this study, the models were retested in version 2024A SP3 (software build 15.1.3.10) at the time the RayStation TPS was upgraded in June 2025. Version 2024A introduced a tree‐view interface unifying them into one combined model covering 70 OARs across 5 anatomical regions. Most individual models were unchanged, but 8 existing OAR models were updated, reflecting improved training‐data segmentation, expanded datasets, and retraining, including: Anorectum, Femur_Head_L/R, HumeralHead_L/R, Prostate, SpinalCord, and Trachea, and 3 new models were added: Prostate_minus_VenousPlexus, SeminalVes, and Spc_Bowel. The training pipeline and execution methods within RayStation were unchanged. This study evaluated 45 OARs that are commonly contoured in clinical routine; other structures the model can segment were excluded since they lack a validated clinical ground truth and are not used clinically. Approximate DLS processing times in RayStation 2024A were 1 min 50 s for thorax (6 organs), 35 s for male pelvis (5 organs), 1 min 18 s for head and neck (16 organs), 45 s for breast (7 organs), and 47 s for abdomen (5 organs).

### 2.3 Regression tests

Regression testing was performed after an update to a previously commissioned software version or DLS model, whereas newly introduced models required initial commissioning. We did not prescribe a fixed number of cases, as sample size should be statistically justified and sufficient to represent relevant clinical and anatomical variability. In this study, the 18–21 cases per anatomical site from the original commissioning were reused for regression testing. All DLS generated in RayStation 2024A were compared with the corresponding clinical segmentations used as the reference standard. Segmentation performance was evaluated using geometric and dosimetric metrics. For head and neck, thorax, and male pelvis, results were compared with the initial commissioning in RayStation 11B to assess version‐related changes and continued clinical suitability. Breast models were re‐evaluated geometrically for potential improvement, whereas the abdominal model was commissioned for the first time. Paired comparison plots and paired *t*‐tests (*α* = 0.05) were used to evaluate geometric differences between software versions.

### 2.4 Evaluation metrics

#### 2.4.1 Geometric evaluation metrics

Segmentation performance was evaluated using the Dice similarity coefficient (DSC), the 95% Hausdorff distance (HD95), and the mean surface distance (MSD). DSC measures the volumetric overlap between the DLS and reference segmentations, with higher values indicating better agreement. HD95 quantifies the 95th percentile of the maximum surface distance between contours, while MSD measures the average surface distance between contours; lower HD95 and MSD values indicate better segmentation accuracy.^19^, ^20^

#### 2.4.2 Dosimetric evaluation metrics

The dosimetric evaluation assessed how DLS versus clinical segmentation deviations affect actual dose to specific OARs. Using the original clinical plans, DVH data were generated for both DLS and clinical contours, then ΔDmean and ΔDmax were calculated and normalized to each case's prescription dose. Prescription doses ranged 14–69.96 Gy (head and neck), 32–66 Gy (thorax), 24–40 Gy (prostate), and 27–67.5 Gy (abdomen). Breast was excluded, as it wasn't commissioned for clinical use.

## 3 RESULTS

### 3.1 Geometric evaluation

**FIGURE 1:**
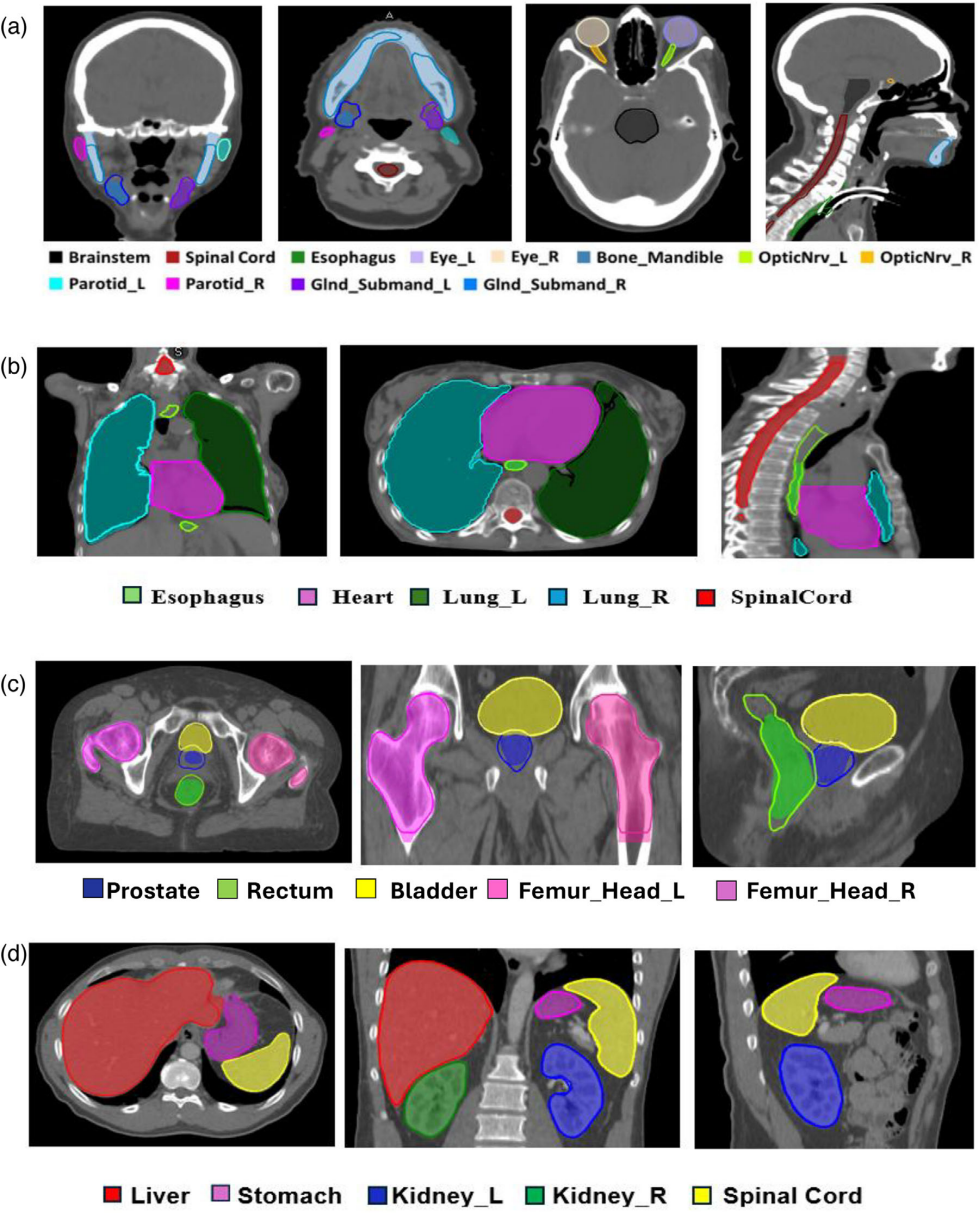
Illustration of deep‐learning segmentation results (contour) in RayStation 2024A compared with the clinical segmentation (colorwash) for (a) a typical head and neck case, (b) a typical thoracic case, (c) a typical male pelvis case, and (d) a typical abdominal case.

Geometric indices were compared among the DLS segmentations in RayStation 11B and RayStation 2024A against the clinical segmentations across all anatomic sites. Quantitative results in terms of the DSC, HD95, and MSD metrics are shown in Table 1. Examples of qualitative comparisons between DLS and clinical segmentations for 4 anatomic sites are illustrated in Figure 1.

**TABLE 1:** Geometric evaluation of structures created by DLS in RayStation 11B and RayStation 2024A, both compared against clinical segmentation.

	RayStation 11B			RayStation 2024A			*P* Values		
ROIs	DSC	HD95, mm	MSD, mm	DSC	HD95, mm	MSD, mm	DSC	HD95	MSD
**Head & Neck Structures** ^1^									
Brainstem	0.89 ± 0.03	4.29 ± 2.00	1.05 ± 0.35	0.89 ± 0.03	4.76 ± 2.29	1.14 ± 0.43	0.39	0.25	0.46
Cochlea_L	0.66 ± 0.14	2.90 ± 1.22	0.70 ± 0.36	0.69 ± 0.15	2.75 ± 1.36	0.62 ± 0.39	0.07	0.29	0.06
Cochlea_R	0.68 ± 0.11	2.61 ± 0.93	0.62 ± 0.27	0.71 ± 0.12	2.69 ± 1.08	0.57 ± 0.29	**0.03**	0.51	0.07
Eye_L	0.91 ± 0.03	2.35 ± 0.53	0.52 ± 0.20	0.92 ± 0.03	2.23 ± 0.65	0.47 ± 0.20	0.12	0.55	0.10
Eye_R	0.92 ± 0.02	2.11 ± 0.77	0.46 ± 0.20	0.93 ± 0.02	1.99 ± 0.70	0.39 ± 0.16	**0.01**	0.42	**0.03**
Lens_L	0.80 ± 0.13	1.99 ± 0.61	0.37 ± 0.21	0.79 ± 0.13	2.07 ± 0.63	0.37 ± 0.21	0.38	0.56	0.33
Lens_R	0.80 ± 0.08	1.88 ± 0.59	0.35 ± 0.14	0.80 ± 0.10	1.80 ± 0.56	0.35 ± 0.15	0.76	0.31	0.52
Bone_Mandible	0.87 ± 0.02	11.05 ± 4.24	1.15 ± 0.31	0.89 ± 0.02	9.36 ± 3.83	0.99 ± 0.24	**0.02**	**0.01**	**0.01**
OpticNrv_L	0.66 ± 0.07	6.62 ± 2.73	1.01 ± 0.39	0.65 ± 0.07	7.36 ± 5.08	1.10 ± 0.61	0.70	0.68	0.72
OpticNrv_R	0.63 ± 0.10	7.27 ± 2.97	1.08 ± 0.43	0.66 ± 0.07	7.41 ± 4.08	1.08 ± 0.49	0.22	0.73	0.76
Parotid_L	0.86 ± 0.05	7.70 ± 5.37	1.41 ± 1.11	0.86 ± 0.04	7.70 ± 6.97	1.41 ± 1.02	0.80	0.74	0.74
Parotid_R	0.88 ± 0.02	5.66 ± 2.95	1.01 ± 0.32	0.87 ± 0.03	5.54 ± 2.86	1.13 ± 0.44	0.07	0.79	0.11
SpinalCord	0.86 ± 0.04	5.22 ± 8.82	0.71 ± 0.56	0.88 ± 0.03	4.25 ± 7.77	0.60 ± 0.53	**0.01**	0.67	0.46
Glnd_Submand_L	0.80 ± 0.12	4.60 ± 2.68	1.10 ± 0.70	0.79 ± 0.17	4.91 ± 3.90	1.16 ± 1.08	0.61	0.65	0.62
Glnd_Submand_R	0.79 ± 0.16	4.70 ± 3.55	1.08 ± 0.86	0.80 ± 0.16	4.77 ± 3.94	1.06 ± 0.95	0.17	0.79	0.76
Esophagus	0.84 ± 0.07	4.73 ± 4.84	0.79 ± 0.67	0.83 ± 0.05	6.40 ± 4.35	0.91 ± 0.43	0.46	0.29	0.57

Thorax Structures^2^									
Lung_L	0.95 ± 0.02	8.20 ± 3.47	1.53 ± 0.70	0.94 ± 0.02	9.17 ± 4.30	1.70 ± 0.79	**0.03**	0.08	**0.02**
Lung_R	0.93 ± 0.03	13.34 ± 7.43	2.51 ± 1.15	0.93 ± 0.03	12.76 ± 6.66	2.42 ± 1.12	**0.04**	0.19	0.08
Heart	0.92 ± 0.03	9.12 ± 4.20	2.71 ± 1.20	0.92 ± 0.04	8.70 ± 5.70	2.72 ± 1.53	0.69	0.59	0.92
Esophagus	0.77 ± 0.12	9.08 ± 9.13	1.77 ± 1.70	0.79 ± 0.10	7.23 ± 8.20	1.42 ± 1.33	0.07	**0.00**	**0.02**
SpinalCord vs. SpinalCord	0.80 ± 0.06	3.20 ± 0.77	1.06 ± 0.36	0.59 ± 0.17	10.19 ± 19.12	5.86 ± 15.75	**0.00**	0.14	0.21
SpinalCanal vs. SpinalCord	**0.87 **± 0.05	2.56 ± 1.20	0.67 ± 0.42	**0.87 **± 0.06	2.64 ± 1.36	0.68 ± 0.42	0.93	0.88	0.91

Male Pelvic Structures^3^									
Prostate	0.77 ± 0.09	10.43 ± 5.01	2.79 ± 1.15	0.80 ± 0.06	7.84 ± 4.10	2.19 ± 0.72	0.06	0.06	**0.035**
Rectum	0.81 ± 0.08	17.47 ± 10.95	2.85 ± 1.72	0.83 ± 0.05	16.42 ± 6.02	2.54 ± 0.93	0.39	0.73	0.468
Bladder	0.96 ± 0.02	6.80 ± 10.54	0.96 ± 0.95	0.96 ± 0.02	4.16 ± 3.49	0.78 ± 0.53	0.39	0.20	0.262
Femur_Head_L	0.90 ± 0.04	22.77 ± 13.79	2.19 ± 1.19	0.91 ± 0.03	21.57 ± 11.56	1.86 ± 0.94	0.41	0.67	0.165
Femur_Head_R	0.91 ± 0.03	22.03 ± 12.87	2.07 ± 1.06	0.93 ± 0.02	16.05 ± 8.09	1.41 ± 0.59	0.40	0.65	0.162

*P* values are derived from paired *t* tests of the indicated geometric metrics.

Abbreviations: DSC, Dice similarity coefficient; HD95, 95% Hausdorff distance; MSD, mean surface distance; ROI, regions of interest.

^1^Values are means ± standard deviations for treatment plans for 18 head‐and‐neck CT datasets.

^2^Values are means ± standard deviations for treatment plans for 18 thoracic datasets. [At our institution, the clinical structure labeled “SpinalCord” represents the contoured spinal canal rather than the anatomical spinal cord. Therefore, both DLS SpinalCord and DLS SpinalCanal were evaluated against this clinical spinal canal contour (labeled as “SpinalCord” in this table).].

^3^Values are means ± standard deviations of treatment plans for 18 men with pelvic CT datasets.

#### 3.1.1 Head and neck

For major head‐and‐neck structures, DSC values remained high in RayStation 2024A compared with the previous version (RayStation 11B). DSC values for well‐defined structures such as the brainstem, spinal cord, and bilateral parotid glands were all ≥0.85 (Table 1, Figure S1). Some small structures (cochleae, optic nerves) showed lower DSC values in the 0.65–0.75 range and larger HD95 values, consistent with greater local boundary uncertainty. The updated model modestly improved segmentation of several structures, including the mandible (DSC from 0.87 to 0.89, with HD95 reduced from 11.1 mm to 9.4 mm) and the eyes and cochleae, whereas performance for submandibular glands and esophagus remained unchanged.

#### 3.1.2 Thorax

Lung (DSC≈0.93–0.95) and heart (DSC≈0.92) segmentation stayed high and stable (Table 1, Figure S2). Esophagus improved modestly (DSC 0.77→0.79; HD95 9.1→7.2 mm). At our institution, the clinically used structure labeled “spinal cord” is contoured to represent the spinal canal rather than the anatomical spinal cord itself. Therefore, for this study, the DLS‐generated spinal canal was compared with the institutional clinical contour labeled “spinal cord”; anatomically, both contours represent the spinal canal. The apparently worse spinal cord metrics in 2024A should therefore not be interpreted as a true decline resulting from comparison of different anatomical structures. However, in one patient, the 2024A model failed to correctly segment the spinal canal and instead generated a contour along the chest wall. This represented a true segmentation failure and highlights the importance of case‐level review following model updates.

#### 3.1.3 Male pelvis

Bladder and femoral heads had DSC > 0.90; prostate and rectum were more moderate and variable across patients (Table 1, Figure S3). Version 2024A improved somewhat over 11B, but accuracy still suffered with prostate spacing agents (SpaceOAR, Barrigel), since these alter local anatomy in ways not represented in the DLS training data. Prostate and rectum may still need manual editing, cutting into the time savings from auto‐segmentation.

#### 3.1.4 Abdomen

The abdomen site was first commissioned in this study by using 20 contrast‐enhanced, breath‐hold abdominal CT datasets. Kidneys, liver, and spleen were consistently segmented with high accuracy (DSC > 0.90; Table 2, Figure S4). Stomach delineation was less accurate, with slightly lower DSC and higher variability reflecting its variable shape and filling status.

**TABLE 2:** Geometric evaluation of abdomen structures created by DLS in RayStation 2024A versus clinical segmentation.

ROIs	DSC	95% HD, mm	MSD, mm
Kidney_L	0.95 ± 0.01	4.10 ± 2.93	0.62 ± 0.27
Kidney_R	0.96 ± 0.01	2.73 ± 1.54	0.50 ± 0.16
Liver	0.96 ± 0.01	5.56 ± 3.78	1.24 ± 0.39
Spleen	0.95 ± 0.02	4.23 ± 6.36	0.69 ± 0.63
Stomach	0.89 ± 0.03	11.78 ± 8.58	1.64 ± 0.59

Values are means ± standard deviations for treatment plans from 20 patients with CT datasets of the abdomen.

Abbreviations: DSC, Dice similarity coefficient; HD95, 95% Hausdorff distance; MSD, mean surface distance; ROI: regions of interest.

#### 3.1.5 Breast

The breast DLS model was evaluated in version 11B but was not commissioned for clinical use at that time because of the large discrepancy between DLS and the clinical segmentations, likely due to differences in contouring guidelines and styles between the model training data and the institutional contour data. The same institutional breast patients (10 left‐sided, 11 right‐sided) were used to re‐evaluate the breast DLS model in version 2024A. Again, the results were consistent with the previous version: the updated breast model cannot be used for our routine clinical practice (Table S1). Geometric performance for breast target volumes and nodal regions was highly variable, particularly in the internal mammary and axillary nodal regions. In one right‐sided breast case, the model incorrectly segmented a shoulder as Breast_R target volume, showing the low robustness performance of the DLS model.

### 3.2 Geometry regression test

**FIGURE 2:**
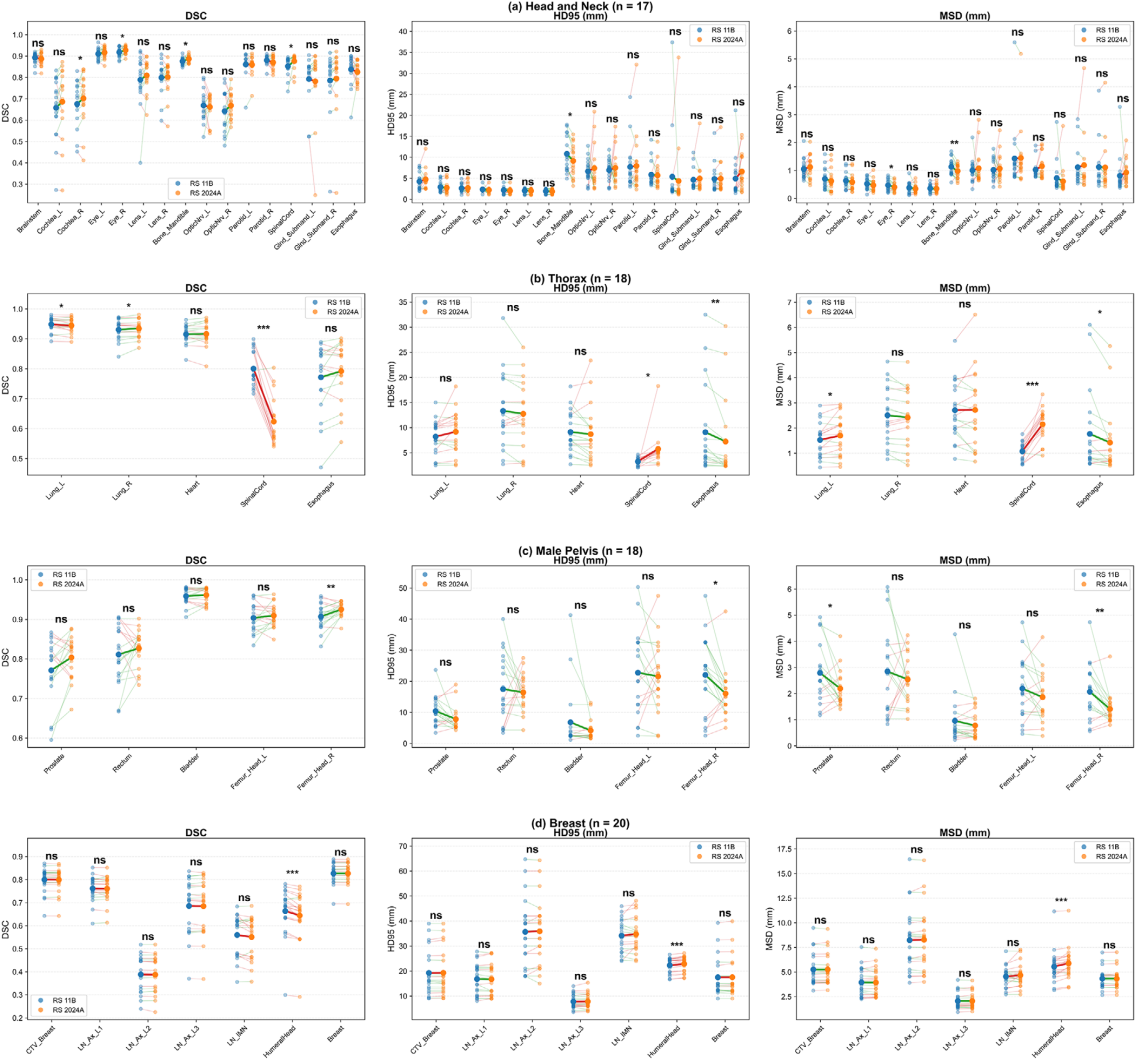
Paired comparison of segmentation performance between RayStation 11B (blue markers) and RayStation 2024A (orange markers). Individual patient results for Dice similarity coefficient (DSC), 95% Hausdorff distance (HD95), and mean surface distance (MSD) are shown for each structure, with lines connecting the same patient in two segmentation methods. Group mean values are indicated by the larger filled markers connected by thick lines. Statistical significance is denoted as *p < 0.05, **p < 0.01), ***p < 0.001), and ns (not significant). (a) Head and neck cohort (*n* = 17), (b) Thorax cohort (*n* = 18), (c) Male pelvis cohort (*n* = 18), (d) Breast cohort (*n* = 20).

Geometric agreement varied primarily with structure characteristics rather than disease site. Large, well‐defined organs generally demonstrated high agreement with clinical segmentations, whereas smaller or less distinct structures showed greater variability. Version‐related differences were selective rather than consistent across sites. As shown in Figure 2, high‐performing structures exhibited minimal differences between RayStation 11B and 2024A, while more challenging structures displayed greater variability, particularly in the breast cohort. Boundary‐based metrics were generally more sensitive than DSC, and cases with incorrect spinal cord and Breast_R segmentations were excluded from Figure 2.

In the head‐and‐neck cohort, statistically significant differences were observed for Bone_Mandible across all geometric metrics, Cochlea_R DSC (*p *= 0.03), and Eye_R MSD (*p *= 0.03). Near‐significant differences were observed for Cochlea_L MSD (*p *= 0.06), but most other structures showed no significant changes (Table 1).

In the thorax cohort, significant improvement was observed for Lung_R DSC (*p *= 0.04), whereas Lung_L showed a significant decrease in DSC (*p *= 0.03) and worse MSD (*p *= 0.02). The esophagus showed significant improvement in HD95 (*p *= 0.00) and MSD (*p *= 0.02). Spinal cord DSC decreased significantly (*p *< 0.001), with the upgraded DLS model in RayStation 2024A failing to generate a correct spinal cord contour in one case. Heart segmentation showed no significant differences between versions (Table 1).

In the male pelvis cohort, the prostate showed significant improvement in MSD (*p *= 0.03), with DSC and HD95 showing near‐significant improvement (*p*≈0.06). No significant differences were observed for bladder, rectum, Femur_Head_R or Femur_Head_L (Table 1).

### 3.3 Dosimetric evaluation

Dosimetric impact was assessed via structure‐specific ΔDmax/ΔDmean, reported both as relative differences (normalized to prescription dose) and absolute Gy values (Tables S2, S3), the absolute values matter because normalized differences can look inflated for low‐dose OARs. Site‐level dosimetric summaries and DVH analyses are in the Supplementary Materials (Figures S5–S7). Breast was excluded from dosimetric evaluation since its DLS model wasn't clinically commissioned.

#### 3.3.1 Head and neck

**FIGURE 3:**
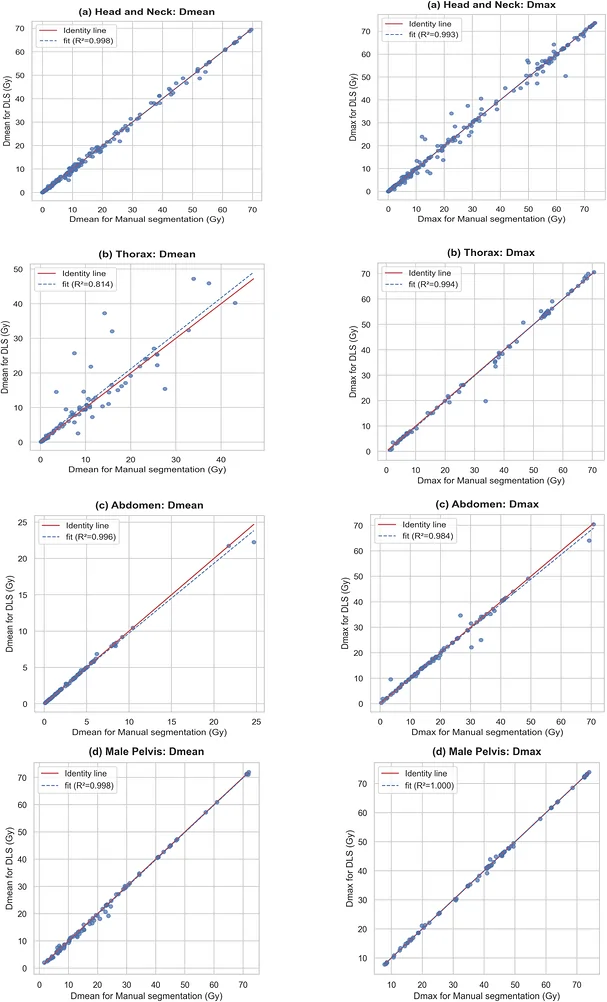
Scatter plots of Dmean (left) and Dmax (right) from deep‐learning segmentation (DLS) in RayStation 2024A versus the clinical segmentation for (a) head and neck, (b) male pelvis, (c) thorax, and (d) abdomen sites. The red line is identity line showing the perfect situation (DLS segmentation equivalent to clinical segmentation) and the blue dashed line is the linear regression fit (*R^2^* ≈ 0.99 for head and neck, male pelvis, and abdomen; *R^2^* ≈ 0.81 for thorax Dmean). The closer the blue dashed line to the red line, the smaller dosimetric impact of inaccurate DLS segmentation.

Normalized ΔDmean was small across OARs: within ± 2% of prescription dose for all structures, and typically < 1% for brainstem, eyes, lenses, optic nerves, spinal cord, and salivary glands. Mean ΔDmax was also near zero, generally within ± 4% (Table S2). The most variability appeared in small, low‐dose structures like the cochleae and optic nerves, with outliers from −17% to +11%, though these corresponded to tiny absolute dose differences. High correlation (*R^2^* = 0.998 Dmean, *R^2^* = 0.993 Dmax) and narrow interquartile ranges confirm excellent dosimetric agreement in the head‐and‐neck region (Table S3, Figure 3a).

#### 3.3.2 Thorax

Lungs and heart showed dose agreement tightly centered near zero: mean ΔDmean was 0.32% (heart), 0.39% (Lung_L), 0.32% (Lung_R), with interquartile ranges roughly ± 1.5%. Mean ΔDmax was similarly small (0.22%, 0.48%, 0.83% respectively), though outliers reached 5.3% (Lung_L), 8.5% (Lung_R), and 4.7% (heart). Spinal cord showed a negative bias mean ΔDmean −3.58%, mean ΔDmax −4.34%, with most cases within ± 5%, but outliers as large as −24% (ΔDmean) and −39% (ΔDmax), tied to geometric labeling mismatches (Tables S2, S3). Esophagus was the biggest outlier: median ΔDmean near 1% (1.01%), but mean 12.28% and SD 16.66%, driven by a small number of cases deviating up to ∼46%. This is related to its poorer geometric accuracy. Visual review found the auto‐segmented esophagus was often truncated, not covering the full superior‐inferior extent of the clinical contour, causing partial organ omission and larger dose errors. Thoracic Dmax was reproduced accurately (*R^2^* = 0.994), but Dmean agreement was weaker (*R^2^* = 0.814, Figure 3b) due to contour discrepancies specifically in the esophagus and spinal cord. This suggests that tubular structures warrant careful human review of contouring extent when using auto‐segmentation.

#### 3.3.3 Male pelvis

Dose agreement was excellent overall. ΔDmean was negligible for anorectum (0.08%) and bladder (0.24%), and near‐zero for prostate (−0.29%, most within ± 1%). Femoral heads showed slightly larger but still modest deviations: −1.46% (left) and −0.77% (right), ranging roughly −7% to +3% (Table S2). ΔDmax stayed within ± 0.3% for all structures (Table S3). Strong correlations (*R^2^* = 0.998 Dmean, *R^2^* = 1.000 Dmax) confirm close DLS‐clinical agreement (Figure 3c).

#### 3.3.4 Abdomen

ΔDmean was very small across all OARs: 0.03%/−0.03% (left/right kidney), −0.04% (liver), 0.16% (spleen), −0.25% (stomach), with interquartile ranges within ± 0.2% (Table S2). ΔDmax averages ranged −2.67% to 0.75%, though outliers appeared, stomach up to ∼22%, liver down to ∼−23% (Table S3). Case‐by‐case visual review of the two notable abdominal Dmax outliers showed that the stomach outlier was associated with under‐contouring by the DLS model, resulting in partial omission of the clinical stomach volume. For the liver outlier, the DLS and clinical contours showed high overall geometric agreement with small, localized boundary differences. Because Dmax is sensitive to a limited number of high‐dose voxels, these localized contour differences may result in a relatively large Dmax difference despite good overall geometric agreement. Strong correlations (*R^2^* = 0.996 Dmean, *R^2^* = 0.984 Dmax) and narrow spreads confirm limited dosimetric impact from geometric differences (Figure 3d).

## 4 DISCUSSION

This study presents a regression testing procedure for RayStation DLS auto‐segmentation models after a software upgrade, using the same patient datasets from initial commissioning for direct comparison. Geometric evaluation (auto‐segmentations vs. clinical contours) and dosimetric analysis were combined to assess clinical acceptability and track performance changes, establishing this as a standard QA step after software or model upgrades.

Across head and neck, thorax, and male pelvis sites, RayStation 2024A showed overall consistent, slightly improved performance versus version 11B, with the biggest gains in the right femoral head, bone mandible, cochleae, and thorax esophagus/lungs, consistent with vendor release notes. Most other structures were stable, but breast segmentation still showed limitations versus local contouring practice, and accuracy was still affected by anatomic complexity (e.g., prostate spacers) and inter‐observer variability.

Geometric agreement (DSC) generally predicted small dosimetric differences (ΔDmean/ΔDmax near zero), but this relationship was not uniform: serial organs in steep dose gradients (spinal cord, especially left lung) showed larger ΔDmax from modest contour discrepancies, while parallel organs (kidneys, liver, salivary glands) stayed dosimetrically robust despite similar DSC variation. This means DSC alone can underestimate clinical impact for high‐gradient OARs, so combined geometric and dosimetric evaluation is needed. By site, dose agreement was strongest for male pelvis, then abdomen, head and neck, and thorax (Dmax *R^2^* = 1.000, 0.984, 0.993, 0.994 respectively), with Dmean showing a similar strong‐correlation pattern.

Our study has some limitations. First, single institutions, CT‐based data with local contouring conventions that may not match the practice of other centers; therefore, the test results may not be directly adopted by other centers. Second, clinical contours are subject to inter‐observer variability and center specific. Third, the test cases were typical or normal anatomy, so DLS failure modes on unusual anatomy were not captured in this study. Finally, because clinical reference contours are subject to inter‐observer variability and no consensus exists on structure‐specific metric thresholds for commissioning, these results should not be interpreted as a universal pass/fail decision rule for clinical use. Rather, the framework proposes a commissioning procedure, characterizes the strengths and weaknesses of the DLS tool, and identifies structures that warrant closer review. Each auto‐segmented structure must therefore be reviewed by the treating physician, who decides whether to accept it, edit it, or discard it and segment the structure from scratch.

The intended use of the evaluated DLS models is for routine clinical CT images and typical anatomy similar to the cases included in this study. Unusual anatomy, substantial imaging artifacts, and postoperative anatomical changes were not specifically evaluated; therefore, model performance under these conditions remains uncertain and warrants careful clinical review. In addition, prostate spacers, such as SpaceOAR and Barrigel, may alter local anatomy and affect prostate and rectum segmentation accuracy. DLS‐generated contours under these conditions should be carefully reviewed and manually edited or replaced when necessary.

Rare but potentially consequential segmentation failures should be evaluated and reported separately from cohort‐averaged geometric and dosimetric results, as favorable summary metrics may not reveal isolated case‐level errors. In this study, the observed failure cases and their frequencies were explicitly reported. Any anatomically implausible or potentially clinically significant failure identified during commissioning, regression testing, or routine clinical use should be documented, communicated to clinical users, and reported to the vendor for investigation and potential corrective action.

## 5 CONCLUSIONS

We presented a practical QA framework for initial commissioning and regression testing after software or model upgrades that may be adapted by other institutions according to their clinical practices and locally validated datasets. Our study underscores the need for site‐ and structure‐specific validation before clinical use, and recommends institutions keep their own benchmark datasets for ongoing performance monitoring.

## AUTHOR CONTRIBUTIONS

**Roya Barati**: Conceptualization; methodology; software; formal analysis; data curation; visualization; writing—original draft. **Jingwei Duan**: Methodology; formal analysis; writing—review and editing. **Song Gao**: Software; data curation; review and editing. **Yao Zhao**: Validation; review and editing. **Xinru Chen**: Data curation; review and editing. **Cenji Yu**: Validation; writing—review and editing. **Zhan Xu**: Validation; writing—review and editing. **Moghadaseh Khaleghibizaki**: Validation; writing—review and editing. **Fredrik Lofman**: Validation; writing—review and editing. **Jared Ohrt**: Writing—review and editing. **Laurence Court**: Resources; review and editing. **Peter Balter**: Supervision; resources; review and editing. **Jinzhong Yang**: Conceptualization; methodology; supervision; project administration; writing—review and editing. All authors reviewed and approved the final manuscript.

## CONFLICT OF INTEREST STATEMENT

The authors declare no conflicts of interest.

## ETHICS AND IRB COMPLIANCE STATEMENT

This retrospective study was approved by the institutional review board of UT MD Anderson Cancer Center (protocol # RCR03‐0400) and conducted in accordance with the Declaration of Helsinki. The requirement for informed consent was waived owing to the retrospective study design.

## Supporting information



Supporting Information

## Data Availability

The data that supports the findings of this study are available from the corresponding author upon reasonable request, subject to institutional and applicable privacy restrictions.

## References

[1] Whitfield GA , Price P , Price GJ , Moore CJ . Automated delineation of radiotherapy volumes: are we going in the right direction?. Br J Radiol. 1021;86:20110718‐20110718. doi:10.1259/bjr.20110718 PMC3615399 23239689

[2] Karaca S . Time matters: a review of current radiotherapy practices and efficiency strategies. Technol Cancer Res Treat. 2025;24:15330338251345376. doi:10.1177/15330338251345376 PMC12099146 40398660

[3] Sharma D , Singh G , Burela N , Gayen S , Aishwarya G , Nangia S . Geometric and dosimetric evaluation of a raystation deep learning model for auto‐segmentation of organs at risk in a real‐world head and neck cancer dataset. Clin Oncol. 2025;41:103796. doi:10.1016/j.clon.2025.103796 40120536

[4] Van Den Bosch L , Van Der Schaaf A , Van Der Laan HP , et al. Comprehensive toxicity risk profiling in radiation therapy for head and neck cancer: a new concept for individually optimised treatment. Radiother Oncol. 2021;157:147‐154. doi:10.1016/j.radonc.2021.01.024 33545258

[5] Vorwerk H , Zink K , Schiller R , et al. Protection of quality and innovation in radiation oncology: the prospective multicenter trial the German society of radiation oncology (DEGRO‐QUIRO study): evaluation of time, attendance of medical staff, and resources during radiotherapy with IMRT. Strahlenther Onkol. 2014;190(5):433‐443. doi:10.1007/s00066-014-0634-0 24595416

[6] Wong J , Fong A , McVicar N , et al. Comparing deep learning‐based auto‐segmentation of organs at risk and clinical target volumes to expert inter‐observer variability in radiotherapy planning. Radiother Oncol. 2020;144:152‐158. doi:10.1016/j.radonc.2019.10.019 31812930

[7] Francolini G , Desideri I , Stocchi G , et al. Artificial intelligence in radiotherapy: state of the art and future directions. Med Oncol. 2020;37(6):50. doi:10.1007/s12032-020-01374-w 32323066

[8] Wang C , Zhu X , Hong JC , Zheng D . Artificial intelligence in radiotherapy treatment planning: present and future. Technol Cancer Res Treat. 2019;18:1533033819873922. doi:10.1177/1533033819873922 PMC6732844 31495281

[9] Zhang J , Cui Z , Shi Z , et al. A robust and efficient AI assistant for breast tumor segmentation from DCE‐MRI via a spatial‐temporal framework. Patterns. 2023;4(9):100826. doi:10.1016/j.patter.2023.100826 PMC10499873 37720328

[10] Ronneberger O , Fischer P , Brox T , U‐Net: convolutional Networks for Biomedical Image Segmentation. In: Navab N , Hornegger J , Wells WM , Frangi AF , eds. Medical Image Computing And Computer‐Assisted Intervention—Miccai 2015. 9351. Lecture Notes in Computer Science. Springer International Publishing; 2015:234‐241. doi: 10.1007/978-3-319-24574-4_28

[11] Hüllermeier E , Waegeman W . Aleatoric and epistemic uncertainty in machine learning: an introduction to concepts and methods. Mach Learn. 2021;110(3):457‐506. doi:10.1007/s10994-021-05946-3

[12] Chen X , Zhao Y , Baroudi H , et al. Comparison of vendor‐pretrained and custom‐trained deep learning segmentation models for head‐and‐neck, breast, and prostate cancers. Diagnostics. 2024;14(24):2851. doi:10.3390/diagnostics14242851 PMC11675285 39767212

[13] Sherer MV , Lin D , Elguindi S , et al. Metrics to evaluate the performance of auto‐segmentation for radiation treatment planning: a critical review. Radiother Oncol. 2021;160:185‐191. doi:10.1016/j.radonc.2021.05.003 PMC9444281 33984348

[14] Baroudi H , Brock KK , Cao W , et al. Automated contouring and planning in radiation therapy: what is ‘clinically acceptable’?. Diagnostics. 2023;13(4):667. doi:10.3390/diagnostics13040667 PMC9955359 36832155

[15] Hurkmans C , Bibault JE , Brock KK , et al. A joint ESTRO and AAPM guideline for development, clinical validation and reporting of artificial intelligence models in radiation therapy. Radiother Oncol. 2024;197:110345. doi:10.1016/j.radonc.2024.110345 38838989

[16] Bosma LS , Hussein M , Jameson MG , et al. Tools and recommendations for commissioning and quality assurance of deformable image registration in radiotherapy. Phys Imaging Radiat Oncol. 2024;32:100647. doi:10.1016/j.phro.2024.100647 PMC11424976 39328928

[17] Çiçek Ö , Abdulkadir A , Lienkamp SS , Brox T , Ronneberger O . 3D U‐Net: learning dense volumetric segmentation from sparse annotation. Medical Image Computing and Computer‐Assisted Intervention ‐ MICCAI 2016. 2016;9901:424‐432. doi:10.1016/10.1007/978-3-319-46723-8_49

[18] Kingma DP , Adam BaJ . A method for stochastic optimization. arXiv. 2014. doi:10.48550/ARXIV.1412.6980. Preprint posted online.

[19] Yang J , Veeraraghavan H , Armato SG , et al. Autosegmentation for thoracic radiation treatment planning: a grand challenge at AAPM 2017. Med Phys. 2018;45(10):4568‐4581. doi:10.1002/mp.13141 PMC6714977 30144101

[20] Taha AA , Hanbury A . Metrics for evaluating 3D medical image segmentation: analysis, selection, and tool. BMC Med Imaging. 2015;15:29. doi:10.1186/s12880-015-0068-x PMC4533825 26263899

