# Supplementary material for: Clinical evaluation and regression test of a commercial deep‐learning auto‐segmentation model
Source: J Appl Clin Med Phys. 2026 Sep 23;27(10):e70824. doi: 10.1002/acm2.70824 (PMC13601754; doi:10.1002/acm2.70824)
Supplement: Supplementary file 1 [file ACM2-27-e70824-s001.docx]

## Supplementary Materials

**Figure S1.** Box plots of segmentation performance for structures in the head and neck region, showing: (a) Dice similarity coefficient (DSC), (b) 95th percentile Hausdorff distance (HD95), and (c) mean surface distance (MSD) between the deep-learning segmentations in RayStation 2024A and clinical segmentations. Red lines represent medians; boxes span the interquartile range (25th to 75th percentile); dots indicate outliers.

**Figure S2.** Box plots of segmentation performance for structures in the thorax region, showing: (a) Dice similarity coefficient (DSC), (b) 95th percentile Hausdorff distance (HD95), and (c) mean surface distance (MSD) between the deep-learning segmentations (DLS) in RayStation 2024A and clinical segmentations. Red lines represent medians; boxes span the interquartile range (25th to 75th percentile); dots indicate outliers. Note: this figure shows DLS SpinalCord versus the SpinalCord clinical segmentations.

**Figure S3.** Box plots of segmentation performance for structures in the male pelvis region, showing: (a) Dice similarity coefficient (DSC), (b) 95th percentile Hausdorff distance (HD95), and (c) mean surface distance (MSD) DLS in RayStation 2024A and clinical segmentations. Red lines represent medians; boxes span the interquartile range (25th to 75th percentile); dots indicate outliers.

**Figure S4.** Box plots of segmentation performance for structures in the abdomen region, showing: (a) Dice similarity coefficient (DSC), (b) 95th percentile Hausdorff distance (HD95), and (c) mean surface distance (MSD) between the DLS in RayStation 2024A and clinical segmentations. Red lines represent medians; boxes span the interquartile range (25th to 75th percentile); dots indicate outliers.

**Figure S5.** Boxplots of absolute differences in mean dose (ΔDmean, Gy) DLS in RayStation 2024A and clinical segmentation for all structures in (a) head and neck, (b) thorax, (c) abdomen, and (d) male pelvis sites. Positive values indicate higher dose with DLS relative to clinical segmentations. Red lines represent medians; boxes span the interquartile range (25th to 75th percentile); dots indicate outliers.

**Figure S6.** Boxplots of absolute differences in maximum dose (ΔDmax, Gy) between DLS in RayStation 2024A and clinical segmentations for all structures in (a) head and neck, (b) thorax, (c) abdomen, and (d) male pelvis sites. Positive values indicate higher dose with DLS relative to clinical segmentations. Red lines represent medians; boxes span the interquartile range (25th to 75th percentile); dots indicate outliers.

(a)

(b)

(d)

(c)

**Figure S7.** Comparison of dose-volume histograms between DLS in RayStation 2024A (dashed lines) and clinical segmentations (solid lines) for structures in a representative patient with (a) head and neck, (b) thorax, (c) male pelvis, and (d) abdomen cancer. The DLS compared with clinical segmentations for these cases are illustrated in Figure 1. The high consistency between dashed lines and solid lines indicates high dosimetry agreement between DLS and clinical segmentations.

**Table S1.** Evaluation of segmentation performance for breast cases via Dice similarity coefficient (DSC), 95% Hausdorff distance (HD95), and mean surface distance (MSD) between deep-learning segmentations (DLS) and clinical segmentations for both DLS models in RayStation 11B and RayStation 2024A. Results are shown as means ± standard deviations from 10 patients with left-sided breast cancer and 11 with right-sided breast cancer. ROIs, regions of interest.

|  | ROIs | RayStation 11B | | | | RayStation 2024A | | |
| --- | --- | --- | --- | --- | --- | --- | --- | --- |
|  |  | **DSC** | **HD95, mm** | **MSD, mm** | **Dice** | | **HD95, mm** | **MSD, mm** |
| Left Breast Cases | CTV_Breast_L | 0.87±0.08 | 16.01±10.4 | 3.55±2.93 | 0.83±0.04 | | 17.17±11.66 | 6.87±8.61 |
|  | LN_Ax_L1 | 0.83±0.05 | 10.54±5.43 | 2.33±0.94 | 0.73±0.05 | | 20.43±5.72 | 4.77±1.31 |
|  | LN_Ax_L2 | 0.67±0.08 | 6.28±1.9 | 1.66±0.61 | 0.40 ± 0.07 | | 37.46±10.31 | 8.50 ± 2.32 |
|  | LN_Ax_L3 | 0.69±0.04 | 7.05±2.58 | 1.81±0.28 | 0.74 ± 0.09 | | 7.78 ±3.36 | 1.83 ± 0.74 |
|  | LN_IMN | 0.7±0.08 | 6.35±5.15 | 1.19±0.61 | 0.51 ± 0.09 | | 36.60±4.65 | 4.96 ± 0.88 |
|  | HumeralHead | 0.82±0.11 | 11.0±7.82 | 2.27±1.75 | 0.68 ± 0.09 | | 23.00±3.9 | 5.46 ± 0.70 |
|  | Breast_R | 0.91±0.03 | 16.08±8.23 | 2.63±1.01 | 0.75 ± 0.24 | | 51.34±108.31 | 34.60±87.1 |
| Right Breast Cases | CTV_Breast_R | 0.77±0.06 | 23.41±9.89 | 5.9±1.84 | 0.77 ± 0.06 | | 22.20±10.75 | 8.30 ± 8.80 |
|  | LN_Ax_R1 | 0.79±0.03 | 13.64±4.55 | 3.23±0.94 | 0.79 ± 0.03 | | 12.60±5.00 | 4.63 ± 5.26 |
|  | LN_Ax_R2 | 0.38±0.08 | 34.0±14.91 | 7.99±3.68 | 0.38±0.07 | | 32.49±15.69 | 10.79±10.4 |
|  | LN_Ax_R3 | 0.64±0.12 | 7.83±2.19 | 2.27±0.83 | 0.64±0.11 | | 7.45 ± 2.57 | 2.95±2.60 |
|  | LN_IMN | 0.6±0.07 | 32.63±7.02 | 4.29±1.0 | 0.59 ± 0.06 | | 30.99±10.87 | 7.16±9.89 |
|  | HumeralHead | 0.65±0.13 | 21.67±3.09 | 5.63±2.06 | 0.65±0.06 | | 5.63±4.3 | 28.45±10 |
|  | Breast_L | 0.82±0.05 | 20.73±8.84 | 4.45±1.28 | 0.82 ± 0.05 | | 19.55±9.78 | 6.85±3.5 |

**Table S2.** Summary statistics of the percentage difference of the mean dose (ΔDmean) between the DLS in RayStation 2024A and clinical segmentations for structures across all anatomic sites (head and neck, thorax, male pelvis and abdomen).

|  | Structure | mean_ ΔDmean % | std_ ΔDmean % | median_ ΔDmean % | Q1_ ΔDmean% | Q3_ ΔDmean % | min_ ΔDmean % | max_ ΔDmean % |
| --- | --- | --- | --- | --- | --- | --- | --- | --- |
| HN | Bone_Mandible | 2.02 | 2.06 | 2.06 | 1.38 | 3.21 | -1.22 | 7.02 |
|  | Brainstem | 0.09 | 1.36 | -0.17 | -0.31 | 0.15 | -1.35 | 4.78 |
|  | Cochlea_L | -1.42 | 4.13 | -0.14 | -0.81 | -0.02 | -17.17 | 0.44 |
|  | Cochlea_R | -0.29 | 0.57 | -0.16 | -0.36 | -0.05 | -1.35 | 0.93 |
|  | Esophagus | -1.48 | 1.86 | -0.82 | -2.58 | 0.00 | -5.23 | 0.55 |
|  | Eye_L | 0.27 | 0.53 | 0.01 | -0.02 | 0.07 | -0.07 | 1.58 |
|  | Eye_R | 0.24 | 0.75 | 0.00 | -0.02 | 0.04 | -0.32 | 2.56 |
|  | Glnd_Submand_L | -0.25 | 1.04 | -0.27 | -0.61 | -0.05 | -2.21 | 2.20 |
|  | Glnd_Submand_R | 0.03 | 1.22 | -0.03 | -0.43 | 0.31 | -2.61 | 2.53 |
|  | Lens_L | -0.11 | 0.63 | 0.00 | -0.01 | 0.02 | -2.53 | 0.31 |
|  | Lens_R | -0.03 | 0.06 | -0.01 | -0.03 | 0.01 | -0.18 | 0.06 |
|  | OpticNrv_L | 0.59 | 1.24 | 0.04 | 0.00 | 0.44 | -0.50 | 4.48 |
|  | OpticNrv_R | 0.29 | 3.24 | 0.00 | -0.05 | 0.03 | -6.14 | 11.27 |
|  | Parotid_L | 0.42 | 3.39 | -0.18 | -1.89 | 1.50 | -3.78 | 9.72 |
|  | Parotid_R | -0.72 | 2.17 | -0.14 | -1.78 | 0.11 | -6.62 | 3.02 |
|  | SpinalCord | 0.02 | 1.81 | -0.53 | -0.98 | 0.56 | -2.18 | 5.91 |
| Thorax | Esophagus | 12.28 | 16.66 | 1.01 | -0.41 | 26.30 | -6.05 | 45.79 |
|  | Heart | 0.32 | 1.12 | 0.01 | -0.43 | 0.81 | -1.18 | 3.21 |
|  | Lung_L | 0.39 | 1.31 | -0.01 | -0.47 | 1.07 | -1.38 | 3.41 |
|  | Lung_R | 0.32 | 1.34 | 0.05 | -0.09 | 1.32 | -2.91 | 2.93 |
|  | SpinalCord | -3.58 | 8.20 | -1.51 | -5.61 | -0.16 | -24.11 | 14.07 |
| Male Pelvis | Anorectum | 0.08 | 0.34 | 0.00 | 0.00 | 0.00 | 0.00 | 1.44 |
|  | Bladder | 0.24 | 0.97 | 0.22 | 0.08 | 0.70 | -2.35 | 1.81 |
|  | Femur_Head_L | -1.46 | 2.23 | -1.34 | -2.44 | -0.39 | -7.32 | 3.32 |
|  | Femur_Head_R | -0.77 | 1.50 | -0.99 | -1.57 | -0.38 | -3.30 | 3.51 |
|  | Prostate | -0.29 | 0.84 | -0.05 | -0.09 | 0.00 | -3.42 | 0.06 |
| Abdomen | Kidney_L | 0.03 | 0.12 | 0.01 | -0.04 | 0.10 | -0.17 | 0.25 |
|  | Kidney_R | -0.03 | 0.12 | 0.01 | -0.09 | 0.06 | -0.29 | 0.18 |
|  | Liver | -0.04 | 0.17 | -0.04 | -0.11 | 0.06 | -0.38 | 0.32 |
|  | Spleen | 0.16 | 0.40 | 0.03 | 0.01 | 0.11 | -0.07 | 1.66 |
|  | Stomach | -0.25 | 0.96 | 0.00 | 0.00 | 0.00 | -3.60 | 0.76 |

Abbreviations: std, standard deviation, Q1, median, first quartile, Q3, third quartile, min, minimum, max, maximum.

**Table S3.** Summary statistics of the percentage difference of the maximum dose (ΔDmax) between the DLS in RayStation 2024A and clinical segmentations for structures across all anatomic sites (head and neck, thorax, male pelvis and abdomen).

|  | Structure | mean_ ΔDmax % | std_ ΔDmax % | median_ ΔDmax % | Q1_ ΔDmax % | Q3_ ΔDmax % | min_ ΔDmax % | max_ ΔDmax % |
| --- | --- | --- | --- | --- | --- | --- | --- | --- |
| HN | Bone_Mandible | 0.45 | 2.29 | 0.00 | 0.00 | 0.08 | -2.70 | 8.65 |
|  | Brainstem | -0.46 | 2.36 | -0.19 | -0.79 | 0.17 | -7.44 | 4.23 |
|  | Cochlea_L | -1.28 | 4.18 | -0.05 | -1.82 | 0.00 | -10.93 | 9.10 |
|  | Cochlea_R | -0.91 | 4.06 | 0.00 | -0.43 | 0.00 | -13.02 | 7.20 |
|  | Esophagus | -3.20 | 3.94 | -1.59 | -3.98 | -0.05 | -12.85 | 0.11 |
|  | Eye_L | 3.07 | 5.92 | 0.07 | 0.00 | 2.38 | -0.39 | 18.10 |
|  | Eye_R | 0.79 | 2.93 | 0.00 | -0.01 | 0.16 | -0.99 | 12.03 |
|  | Glnd_Submand_L | 0.21 | 1.90 | 0.00 | -0.01 | 0.00 | -2.68 | 5.79 |
|  | Glnd_Submand_R | 0.88 | 4.99 | 0.00 | -0.28 | 1.85 | -11.03 | 11.87 |
|  | Lens_L | -2.02 | 8.50 | 0.00 | -0.08 | 0.00 | -34.97 | 1.45 |
|  | Lens_R | -0.14 | 0.40 | 0.00 | -0.02 | 0.00 | -1.59 | 0.11 |
|  | OpticNrv_L | 2.14 | 7.92 | 0.01 | -0.06 | 0.19 | -2.82 | 32.27 |
|  | OpticNrv_R | 4.11 | 16.78 | 0.00 | -0.03 | 0.07 | -1.42 | 69.19 |
|  | Parotid_L | -1.66 | 6.47 | 0.00 | -2.06 | 0.59 | -19.28 | 12.53 |
|  | Parotid_R | 0.26 | 2.59 | 0.00 | -0.26 | 1.14 | -5.14 | 7.85 |
|  | SpinalCord | 3.30 | 13.18 | 0.37 | 0.00 | 1.21 | -3.33 | 54.14 |
| Thorax | Esophagus | 0.34 | 0.81 | 0.00 | 0.00 | 0.14 | -0.37 | 2.17 |
|  | Heart | 0.22 | 1.98 | 0.00 | -0.54 | 0.54 | -4.16 | 4.67 |
|  | Lung_L | 0.48 | 1.40 | 0.05 | -0.24 | 0.62 | -1.06 | 5.34 |
|  | Lung_R | 0.83 | 2.09 | 0.14 | 0.00 | 0.49 | -0.54 | 8.52 |
|  | SpinalCord | -4.34 | 8.96 | -2.05 | -2.55 | -1.74 | -38.57 | 0.00 |
| Male Plevis | Anorectum | -0.12 | 0.53 | 0.00 | 0.00 | 0.00 | -2.24 | 0.00 |
|  | Bladder | 0.02 | 0.30 | 0.00 | -0.08 | 0.00 | -0.52 | 0.78 |
|  | Femur_Head_L | -0.01 | 0.95 | 0.00 | -0.43 | 0.51 | -2.38 | 1.76 |
|  | Femur_Head_R | -0.21 | 1.31 | -0.30 | -0.59 | 0.00 | -2.41 | 2.92 |
|  | Prostate | -0.19 | 0.26 | -0.07 | -0.48 | 0.00 | -0.57 | 0.24 |
| Abdomen | Kidney_L | 0.14 | 0.46 | 0.00 | -0.09 | 0.28 | -0.38 | 1.10 |
|  | Kidney_R | 0.75 | 4.41 | 0.00 | -0.26 | 0.12 | -2.50 | 16.92 |
|  | Liver | -2.67 | 5.81 | -0.48 | -3.66 | -0.09 | -23.39 | 2.09 |
|  | Spleen | 0.25 | 0.90 | 0.00 | -0.06 | 0.32 | -1.17 | 2.92 |
|  | Stomach | 0.13 | 7.75 | 0.00 | 0.00 | 0.00 | -20.08 | 22.33 |

Abbreviations: std, standard deviation, Q1, median, first quartile, Q3, third quartile, min, minimum, max, maximum.
